# Experimental and Computational Study of Mechanical and Thermal Characteristics of h-BN and GNP Infused Polymer Composites for Elevated Temperature Applications

**DOI:** 10.3390/ma15155397

**Published:** 2022-08-05

**Authors:** Mantesh C. Choukimath, Nagaraj R. Banapurmath, Fahid Riaz, Arun Y. Patil, Arun R. Jalawadi, M. A. Mujtaba, Kiran Shahapurkar, T. M. Yunus Khan, Mishal Alsehli, Manzoore Elahi M. Soudagar, I. M. R. Fattah

**Affiliations:** 1 School of Mechanical Engineering, KLE Technological University, Hubballi 580031, India; 2 Mechanical Engineering Department, Abu Dhabi University, Abu Dhabi P.O. Box 59911, United Arab Emirates; 3 Department of Mechanical Engineering, Faculty of Engineering, University of Malaya, Kuala Lumpur 50603, Malaysia; 4 Department of Mechanical Engineering, School of Mechanical, Chemical and Materials Engineering, Adama Science and Technology University, Adama 1888, Ethiopia; 5 Department of Mechanical Engineering, College of Engineering, King Khalid University, Abha 61421, Saudi Arabia; 6 Mechanical Engineering Department, College of Engineering, Taif University, P.O. Box 11099, Taif 21944, Saudi Arabia; 7 Department of Mechanical Engineering, University Centre for Research & Development, Chandigarh University, Mohali 140413, India; 8 Department of Mechanical Engineering, School of Technology, Glocal University, SH-57, Mirzapur Pole, Saharanpur District, Uttar Pradesh 247121, India; 9 Centre for Technology in Water and Wastewater (CTWW), Faculty of Engineering and IT, University of Technology Sydney, Ultimo, Sydney, NSW 2007, Australia; 10 Department of Mechanical Engineering, College of Engineering, Universiti Tenaga Nasional, Kajang 43000, Malaysia

**Keywords:** polymer material, high-temperature applications, GNP, h-BN, epoxy

## Abstract

Polymer-based nanocomposites are being considered as replacements for conventional materials in medium to high-temperature applications. This article aims to discover the synergistic effects of reinforcements on the developed polymer-based nanocomposite. An epoxy-based polymer composite was manufactured by reinforcing graphene nanoplatelets (GNP) and h-boron nitride (h-BN) nanofillers. The composites were prepared by varying the reinforcements with the step of 0.1 from 0.1 to 0.6%. Ultrasonication was carried out to ensure the homogenous dispersion of reinforcements. Mechanical, thermal, functional, and scanning electron microscopy (SEM) analysis was carried out on the novel manufactured composites. The evaluation revealed that the polymer composite with GNP 0.2 by wt % has shown an increase in load-bearing capacity by 265% and flexural strength by 165% compared with the pristine form, and the polymer composite with GNP and h-BN 0.6 by wt % showed an increase in load-bearing capacity by 219% and flexural strength by 114% when compared with the pristine form. Furthermore, the evaluation showed that the novel prepared nanocomposite reinforced with GNP and h-BN withstands a higher temperature, around 340 °C, which is validated by thermogravimetric analysis (TGA) trials. The numerical simulation model is implemented to gather the synthesised nanocomposite’s best composition and mechanical properties. The minor error between the simulation and experimental data endorses the model’s validity. To demonstrate the industrial applicability of the presented material, a case study is proposed to predict the temperature range for compressor blades of gas turbine engines containing nanocomposite material as the substrate and graphene/h-BN as reinforcement particles.

## 1. Introduction

In today’s research world, considerable interest has been shifted toward polymer composites embedded with nanofillers and microfillers for high-end technological applications [1]. Many nanofillers are currently used as a strength, temperature, and other property-enhancing catalysts: to name a few, Titanium oxide (TiO_2_), Molybdenum disulphide (MoS_2_), Aluminium oxide (Al_2_O_3_), Silicon carbide (SiC) and many more [2,3]. These nanocomposites have proven to enhance the material’s mechanical, physical, thermal, and tribological properties [4,5,6,7]. However, in recent days, h-BN is another such nanofiller that is quite similar in properties to MoS_2_ and graphite [8]. It works at around 900 °C with high thermal stability when compared to MoS_2_ (1150 °C), Tungsten disulphide (1200 °C), and graphite (3000 °C) [9]. Thermal management systems, neutron shielding, sensors, and improving the thermal and mechanical performances of composites are some of the broad application areas of h-BN-based polymer composites. As per recent studies, h-BN is anticipated to generate around USD 900 million by 2023 [10]. In recent years, much consideration has been given to nano h-BN because of its improved properties (e.g., mechanical strength, high thermal resistance, etc.). h-BN is being utilised progressively due to its excellent combination of properties, which consist of a low density (2.27 g/cm^3^), high-temperature steadiness, and chemical inertness (corrosion resistance against acids and molten metals) [11,12,13].

GNPs have tremendous scope for multifunctional properties. GNPs comprise minute stacks of graphene sheets having a platelet structure similar to those observed in carbon nanotubes in the generic planar form [14]. GNPs can enhance mechanical properties such as the stiffness, strength, and surface hardness of the matrix material. Polymer-based composites have multi-facets. Epoxy resin thermoset is well known for its low shrinkage after curing, excellent mechanical properties, high chemical stability, and better adhesive bond strength. In addition, the epoxy polymer has anti-wear behaviour during curing [14,15]. Epoxy resin has a broad application in adhesive, composite, and protective coating.

The dispersion technique is crucial for ensuring nanoparticle (NPs) distribution in the polymer matrix. The maximum dosage of NPs in the holding matrix is limited due to critical issues associated with the dispersion technique. Ultrasonication is one such process used in the dispersion of NPs uniformly in the matrix material [16].

Tsuji et al. [17] have reported the science behind the adhesion between epoxy matrix and filler materials such as h-BN and graphite. Owais et al. [18] have emphasised the synergetic effect of different fillers in the epoxy matrix, which has resulted in the enhanced thermal conductivity and electrical resistivity of epoxy nanocomposites. Shi et al. [19] emphasised the improvement in the thermal conductivity of h-BN infused epoxy-based composites. Sun et al. [20] have focused on the overall design and fabrication of Boron nitride microsphere/epoxy composites with good cross-plane thermal conductivity and lower dielectric properties. Gu et al. [21] and Ren et al. [22] have significantly increased thermal conductivity with respect to h-BN embedded polymer composites.

Zhang et al. [23,24] have reported the failure mechanisms of anisotropic conductive films due to the thermal degradation of epoxy material when subjected to temperatures beyond 300 °C and also emphasised the effects of acrylic adhesive property with respect to bonding parameters.

Chung et al. [25] found that the surface treatment of the h-BN particles using the silane 3-glycidoxypropyltrimethoxysilane (GPTMS) has increased thermal conductivity. Jiang et al. [26] highlighted the synergistic effect of functionalised graphene/boron nitride on the thermal conductivity of polystyrene composites. The authors found an enhancement of up to 20% compared with the plain composite. Ejaz et al. [27] have reported the role of GNPs (lower dosage) in improving the strength parameters of epoxy-based adhesive material.

The literature survey scantly highlights the usage of nanofillers with h-BN and GNPs embedded in an epoxy and polyimide matrix for high-temperature applications. Currently, the existing works do not fulfil the high-temperature conditions catering to generic structural applications. Therefore, the present work focuses on the experimental studies and numerical analysis of epoxy reinforced with h-BN and GNP composites for structural applications by varying the reinforcements with the step of 0.1 from 0.1 to 0.6%. To further validate the benefits of this study, this research proposes a case study that uses a simulation of polyimide reinforced with both h-BN and GNP composites for the design of a compressor blade to demonstrate the industrial applicability of novel composite manufactured. 

The current work focuses on optimal composition development of h-BN/GNP reinforced in epoxy/polyimide substrate. Furthermore, the future scope of work can be considered for building a working prototype to realise applications in real time for thermal and structural domains.

## 2. Material and Methodology

The properties of the GNP and h-BN used in this work are mentioned in Table 1; the GNPs and h-BNs were industrial grades with purity ≥ 99.5% and 99.9%. For the current studies, GNP and h-BN NPs were procured from Nanoshell Pvt. Ltd. Punjab, India. The matrix epoxy (Trade name—Lapox—L12, Hardener—K6) material used for the current work was procured from Atul Pvt. Ltd. Polymer division, Gujarat, India. Throughout the fabrication of polymer nanocomposites, GNP and h-BNs were dispersed uniformly. The most crucial step in combining GNP and h-BNs is diffusion. The ultrasonication technique, ultrasonication duration, and specimen casting procedures were all kept constant in all polymer composite preparations. As shown in Table 2, varying amounts of GNP and h-BNs were incorporated into the polymer composite matrix. The prepared composite solution has undergone an ultrasonication process for 50 min to attain the composite’s uniform dispersion.

The simulation work is carried out using a design modeller (CAD tool) and ANSYS workbench mechanical with version 18.2. The exhaustive simulation work led to an outcome of a compressor blade coating materials at an intermediate temperature range of 400 to 500 °C. 

### 2.1. Preparation of Specimens

**Figure 1:**
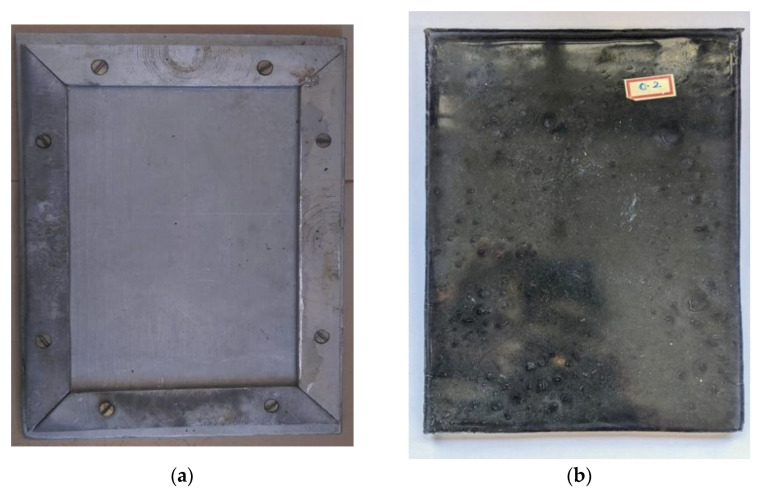
(**a**) Aluminium mould of 230 mm × 164 mm × 6 mm size, (**b**) Epoxy composite plate (0.2 wt % GNP and h-BN).

The GNPs with a purity level of 99.5%, h-BNs with a purity level of 99.9% and a concentration of 0.1%, 0.2%, 0.3%, 0.4%, 0.5% and 0.6% [27] by the total weight of the epoxy matrix was used for the investigation. Two types of composites were prepared. One composite plate was reinforced with only GNP, and the other one was reinforced with GNP and h-BN, as shown in Table 2 and Table 3, in equal proportion. A hardener containing 10% of the weight of plain epoxy matrix was added to start the crystallisation process. To ensure the better dispersion of nanofillers into the parent matrix, all composite mixtures were sonicated for 50 min. The composite was prepared using aluminium moulds with dimensions of 230 mm × 164 mm × 6 mm. The specimens were kept for 24 h of curing. The composite plates were carefully removed from the mould after the curing process. Figure 1a shows the aluminium mould used to prepare composite plates; Figure 1b shows the typical epoxy composite plate (0.2 wt % GNP + h-BN) composite plate sample after removing from the aluminium mould.

#### Testing Standards

Tensile test specimens were prepared according to ASTM D3039. Table 4 shows the details of the ASTM D3039 [28].

The GNP-based composite specimen details utilised for the 3-point bending analysis were prepared as per ASTM (D7264) [29] and shown in Table 5.

The GNP and h-BN based composite specimen details used for the four-point bending test were prepared as per ASTM (D7264) [29] and are shown in Table 6.

### 2.2. Experimental Setup

#### 2.2.1. Tensile Test

The tensile test of the PE and prepared nanocomposites was carried out to determine Young’s Modulus (MPa), tensile strength (MPa), and percentage strain failure. Tensile specimens were cut as per ASTM D3039 standards, and testing was carried out in a Tinius Olsen UTM (10 kN capacity) with fixtures movement at 3 mm/min. In total, four specimens were tested for all the combinations, and the results were averaged.

#### 2.2.2. Flexural Test

The flexural properties of PE and nanocomposites were determined using the three-point bending and four-point bending method; these tests are being performed as per ASTM (D7264) standard, and testing was carried out in a Tinius Olsen UTM (10 kN capacity). The flexural modulus and strength were determined from these tests. In total, four specimens were tested for all the combinations, and the results were averaged. The loading was made until the rupture of the external surface of the sample or until a strain reaches a maximum value of 5%, whichever is earlier. A strain rate of 3 mm/min was set for the testing conditions.

#### 2.2.3. Thermogravimetric Analysis

TGA is a method of thermal analysis in which changes in physical and chemical properties of materials are measured as a function of increasing temperature (with constant heating rate) or as a function of time (with constant temperature and/or constant mass loss). Changes in the mass of a sample due to various thermal conditions are studied while the sample is subjected to a program of change in temperature. The TGA was performed in a nitrogen atmosphere and the specimens were subjected to varying temperatures from 25 to 600 °C with a rise in temperature at 10 °C per minute.

#### 2.2.4. Fourier Transform Infrared Spectroscopy (FTIR)

The elemental composition of the prepared composites was studied by the FTIR route. The mid-infrared region of 400 to 4000 wavenumbers, which equals wavelengths of 2.5 to 25 microns (10^−3^ mm), was used during the test to find out the elemental composition of the prepared specimens.

#### 2.2.5. SEM and Energy-Dispersive X-ray Analysis (EDX)

Scanning Electron Microscopy involves imaging samples with the help of electrons. The large depth of field of SEM allows a large amount of sample to be focused at an instant. SEM analysis is used to study the dispersion of nanoparticles in the polymer matrix. A continuous beam of electrons having a wavelength lower than ordinary light is made to fall on the specimen. The back-reflected electrons from the sample, called secondary electrons, are detected in a detector that prepares the SEM image based on the raster scan technique. Specifications of the machine used for SEM imaging are highlighted in Table 7.

The EDX instrument, which is an optional attachment to the JEOL JSM-63OLA, was used for the elemental analysis. The specifications of the EDX instrument are highlighted in Table 8. 

## 3. Results and Discussion

To comprehend the behaviour of the developed composites, the required quantity of reinforcing agents such as GNP and h-BN in the matrix material was evaluated based on the optimum value of load-bearing capacity, increased ductility, and material degradation of the nanocomposites with temperature rise, the tensile test, 4-point bending, 3-point bending and TGA tests.

### 3.1. Tensile Strength Test

#### 3.1.1. Tensile Test for GNP Based Nanocomposites

**Figure 2:**
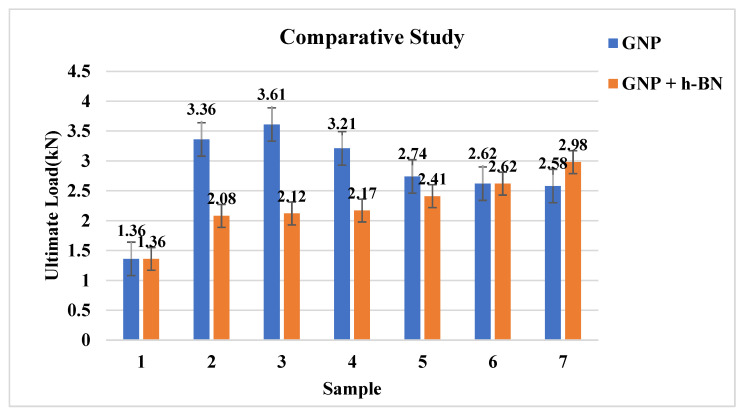
Samples vs. ultimate load of tensile test for composite with GNP and samples vs. ultimate load of tensile test for composite with GNP + h-BN.

This test helps to evaluate the ideal weight percentage of GNP and h-BN for reinforcing with the epoxy matrix for enhancement of the ductile property of the composites. There were in total seven specimens conducted for the tensile strength test; the results were compared between the plain epoxy composite and epoxy nanocomposites reinforced with GNP and h-BN to understand the changes incurred due to reinforcement. Figure 2 shows the variation of the ultimate tensile load for composite reinforced with only GNP. Figure 2 shows that the tensile strength increases as the GNP filler increases. This behaviour is observed until 0.2 wt % of the nanocomposites; this could be due to the homogeneous distribution of GNP fillers in the parent matrix [29], as seen in the SEM micrograph in Figure 8b. The load-bearing capacity of the composite reinforced with GNP (0.2 wt %) increased by 265% compared with the plain epoxy composite.

#### 3.1.2. Tensile Test for GNP and h-BN Based Nanocomposites

Figure 2 also shows the variation of the ultimate tensile load for composite reinforced with GNP and h-BN, respectively. Figure 2 shows that a higher dosage of both GNP and h-BN fillers in the matrix increased the tensile load-bearing capacity. It was observed that with an increase in filler concentration, the composite material’s tensile strength (TS) was increased. The epoxy composite reinforced with GNP and h-BN increased even at a 0.6 weight percentage. The reinforced composite’s load-bearing capacity with GNP and h-BN (0.6 wt %) increased by 219% compared with the plain epoxy composite. However, this behaviour resulted from GNP and h-BN fillers occupying the air pockets, which were open in the pristine epoxy resin. The possible reason for enhancing the results might be the homogenous distribution of fillers in the polymer substrate [29].

### 3.2. Three-Point Bending Test for GNP-Based Composites

**Figure 3:**
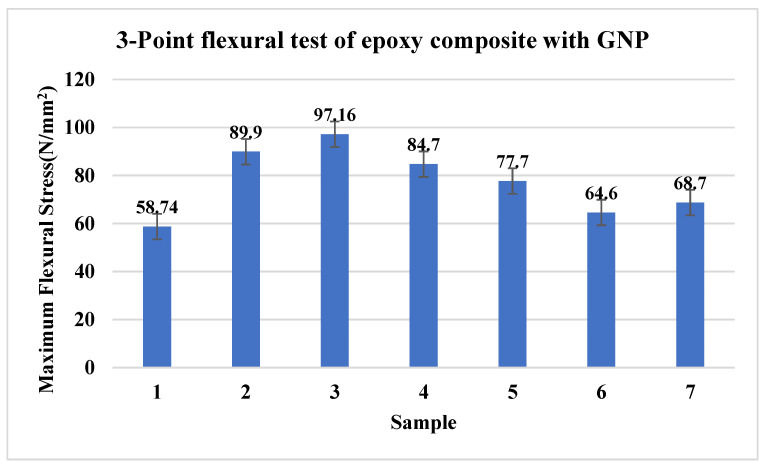
Maximum flexural stress of 3-point bending test.

Figure 3 shows the variation of the maximum flexural stress for composite reinforced with only GNP. Figure 3 shows that a lower dosage of GNP favoured enhanced flexural strength more than the higher dosage, as the latter results in an agglomeration of the nanofillers as seen in SEM micrographs. The developed polymer composite material’s flexural tests show that the addition of GNPs into the polymer matrix increases the polymer composite’s load-bearing capacity as the filler material increases to 0.2 wt % of epoxy. This could be due to the proper dispersion of GNP particles throughout the matrix and the resulting strength of the nanocomposite material. The trend of declination is 33 MPa when compared to 0.5 wt % of GNP. Nevertheless, as the filler material increases, the material’s brittleness will enhance due to the shear yielding and deformation of these filler materials. They form irregular patterns even though they are uniformly spread throughout the polymer composites matrix as the load-bearing capacity of the composites decreases.

### 3.3. Four-Point Bending Test

**Figure 4:**
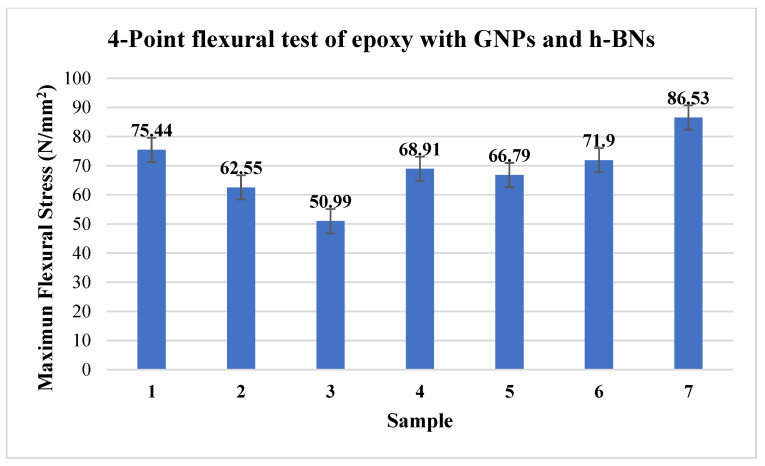
Maximum flexural stress of 4-point bending test.

The four-point bending test provides the modulus of elasticity bending, flexural strain, flexural stress, flexural stress–strain response, and maximum load-bearing capacity of the materials synthesised. Figure 4 shows that maximum flexural stress increased with an increase in the dosage of the GNP and h-BN. The optimum result is obtained at 0.6 wt %. This behaviour is due to the proper dispersion of the nanoparticles in the polymer matrix, as witnessed in the SEM images.

### 3.4. Thermogravimetric Analysis

**Figure 5:**
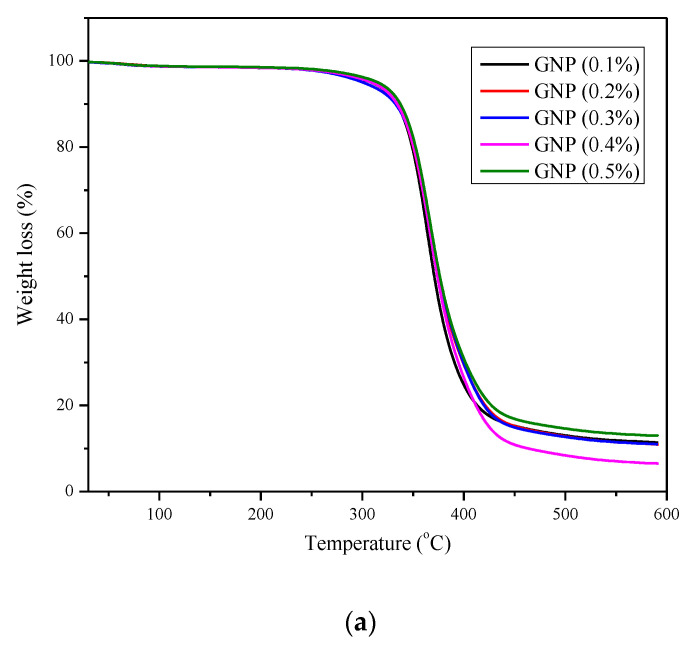

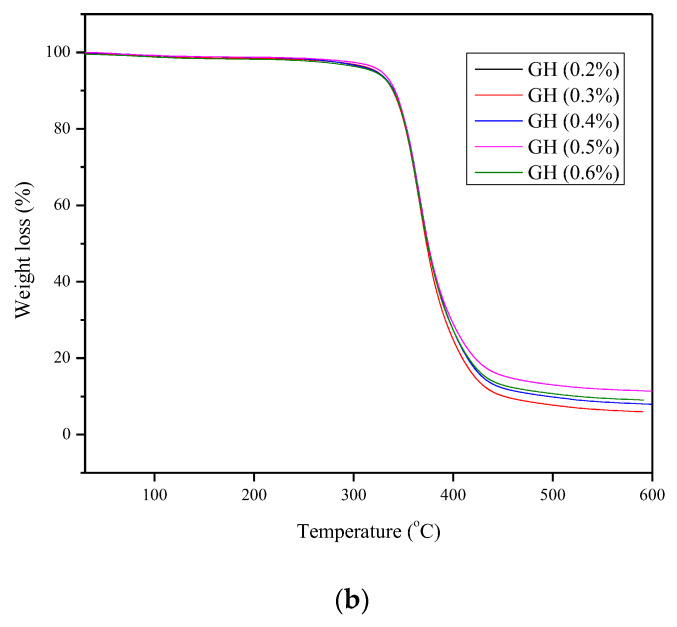
(**a**) TGA test results of nanocomposites reinforced with GNP and (**b**) TGA test results of nanocomposites reinforced with GNP and h-BN.

TGA is performed to study the nanocomposite’s thermal stability. An optimal sample is considered based on the maximum strength of tensile and flexural tests for the brevity of this work. Prepared composites (both GNP and GNP+h-BN based) were subjected to varying temperatures from 25 to 600 °C with a rise in temperature at 10 °C per minute [29]. Figure 5a shows the TGA analysis of nanocomposites reinforced with GNP. Figure 5b shows the TGA analysis of nanocomposites reinforced with a combination of GNP and h-BN. A 1.3% degradation in the mass occurred at a temperature (T_d1.3%_) of around 242 °C for the plain epoxy specimen.

In contrast, the same percentage of mass degradation occurs at a temperature of about 340.99 °C for the polymer composite with 0.2 wt % of GNP and 342.43 °C for polymer reinforced with GNP and h-BN at 0.6 wt %. Thus, there was an increase of 40.9%, i.e., an increase of 99 °C for the polymer composite reinforced with GNP compared to a plain epoxy specimen. Furthermore, an increase of 41.5%, i.e., 101.3 °C for the polymer composite reinforced with GNP and h-BN, is obtained compared to the plain epoxy specimen. A 10% residual at a temperature above 500 °C is observed for the polymer nanocomposites, while plain epoxy showed a comparatively lower temperature [29].

**Figure 6:**
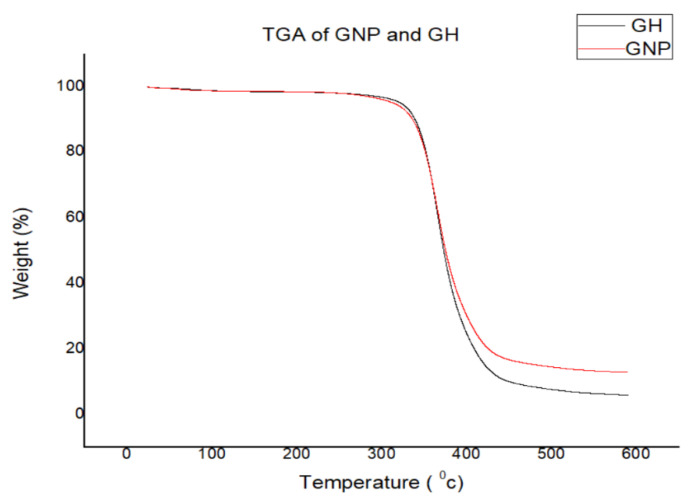
TGA test results of nanocomposites reinforced with optimised wt % of GNP and GH.

Figure 6 shows the TGA test results of nanocomposites reinforced with optimised values wt % of GNP (0.2%) and GH (0.6%). Compared to GH-based composites, GNP filler resulted in higher thermal stability, as is evident from the figure.

### 3.5. Fourier Transform Infrared Spectroscopy

This test helps to understand the bonding between the matrix and filler material used. The FTIR test results of the composites with a varied dosage of nanofillers are carried out. However, for the discussions, an optimal sample is considered based on the higher load-bearing capacity for tensile and flexural test conditions.

**Figure 7:**
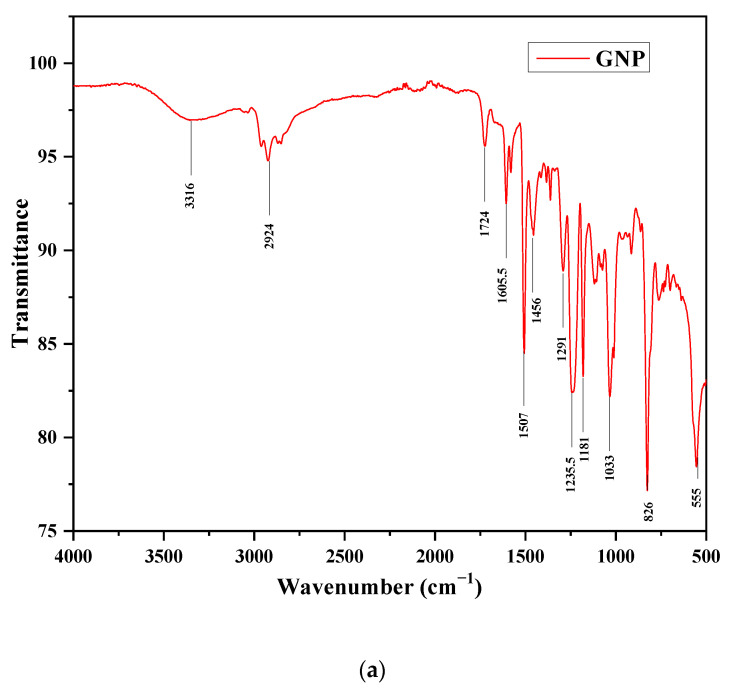

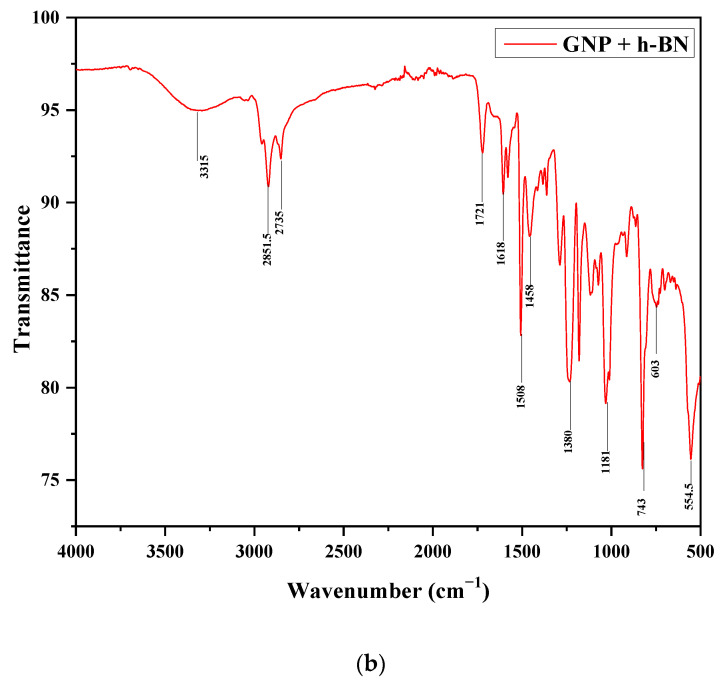
(**a**) FTIR result of the nanocomposite reinforced with GNP (0.2 wt %). (**b**) FTIR result of the composite reinforced with GNP and h-BN (0.6 wt %).

Figure 7a shows the FTIR spectrum of polymer composite reinforced with the optimum GNP quantity, i.e., 0.2 wt % of epoxy composites. The peaks corresponding to the amine N-H stretch appear at 3316 cm^−1^. The peaks at 1507 cm^−1^ correspond to the presence of the nitro compound group. Furthermore, absorption peaks that occur at 826 cm^−1^ confirmed the presence of the aromatic C-H bending group. The FTIR spectrum of polymer composite reinforced with the optimum quantity of GNP and h-BN, i.e., 0.6 weight percentage of epoxy composites, is shown in Figure 7b. The peaks corresponding to the amine N-H stretch appear at 3315 cm^−1^. The peaks at 1508 cm^−1^ correspond to the presence of the nitro compound group. Furthermore, absorption peaks that occur at 743 cm^−1^ confirmed the presence of the aromatic C-H bending group [29].

### 3.6. Scanning Electron Microscope Analysis

The morphology and microstructure of the nanocomposites fracture surface were investigated using an ultrahigh-resolution field emission. SEM is used to investigate the nanoscale interaction of fillers in the holding matrix.

**Figure 8:**
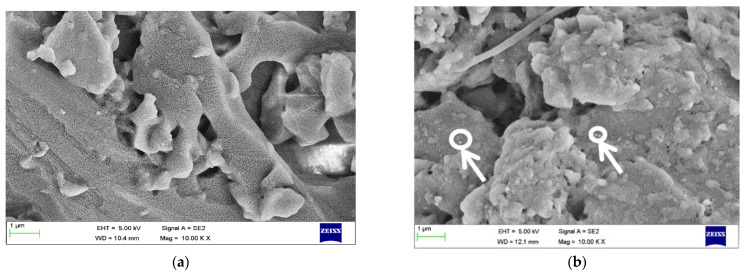
Patterns of nanocomposites with different reinforcement (**a**) 0.1 wt % (**b**) 0.2 wt % of GNP.

Figure 8 shows micrographs of nanocomposites with GNP reinforcement varied from 0.1 to 0.2 wt % of the parent matrix. Figure 8a infers that smaller voids are visible with lower GNP dosage. Figure 8b shows a uniform distribution of GNP resulting in the formation of interconnected layers, with minimal voids resulting in enhanced mechanical properties [30].

**Figure 9:**
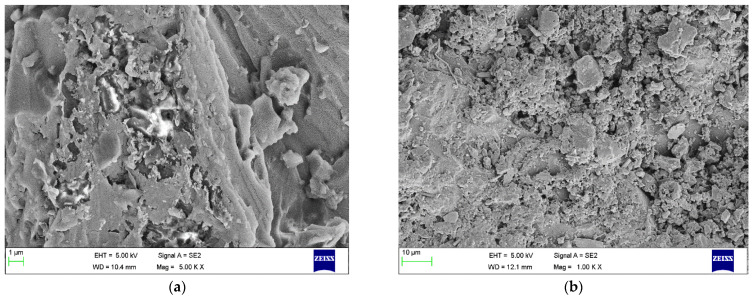
Patterns of nanocomposites with different reinforcement (**a**) 0.5 wt % (**b**) 0.6 wt % of GNP and h-BN.

Figure 9 shows that the micrographs of nanocomposites reinforced with GNP and h-BN nanoparticles varied from 0.5 to 0.6 wt % of the parent matrix. From Figure 9a, an agglomeration of the filler material in localised regions was observed. The fractured surface shows an intergranular behaviour of failure, which usually occurs in metal-based compounds. Increasing the dosage of GNP and h-BN from 0.5 to 0.6 wt % in the matrix shows a homogeneous distribution of the fillers throughout the entire substrate. The failure of a composite with a filler material combination results in brittle fracture, similar to a concrete control mix [31].

The EDX analysis validates the number of elemental compounds present in the prepared composites. Table 9 and Table 10 show the elements and the weight percentage of the specimen used. Elements C and O correspond to the epoxy and GNP constituents, and B and N correspond to the h-BN based composite. The combination of GNP and h-BN at 0.6 wt % has enhanced mechanical properties concerning strength and stiffness; this might be due to the percentage rise in boron, nitrogen and oxygen compared to 0.4 wt % h-BN with epoxy resin.

## 4. Simulation of Polymer-Based Nanocomposites

Industry internet of things (IIOT) has come to the limelight in the last few years with most care for simulation in the design field [32]. The simulation covers software tools with a background such as the finite element method, molecular dynamics, and solid mechanics. Ansys, J-OCTA, Material studio and current software tools are used to analyse the newer materials developed [33]. In this work, the ANSYS workbench is considered as a tool to validate the experimental results.

This section validates the mechanical properties such as total deformation and Von Mises stress of the epoxy-based nanocomposites using a combination of h-BN and GNP as fillers. The lower TGA of epoxy-based nanocomposites demands a higher TGA-based matrix material for high-temperature applications such as compressor blades and internal combustion parts. Polyimide-based nanocomposites were developed for this high-temperature application using ANSYS-based simulation, and the results were predicted solely by numerical methods.

Section 4.1 discusses the numerical results on the epoxy-based nanocomposites reinforced with h-BN and GNP combinations and validation with the experimental work.

Section 4.2 discusses the numerical results of the polyimide-based nanocomposites reinforced with hBN and GNP. The result was validated using the convergence theory.

### 4.1. Simulation Method

**Figure 10:**
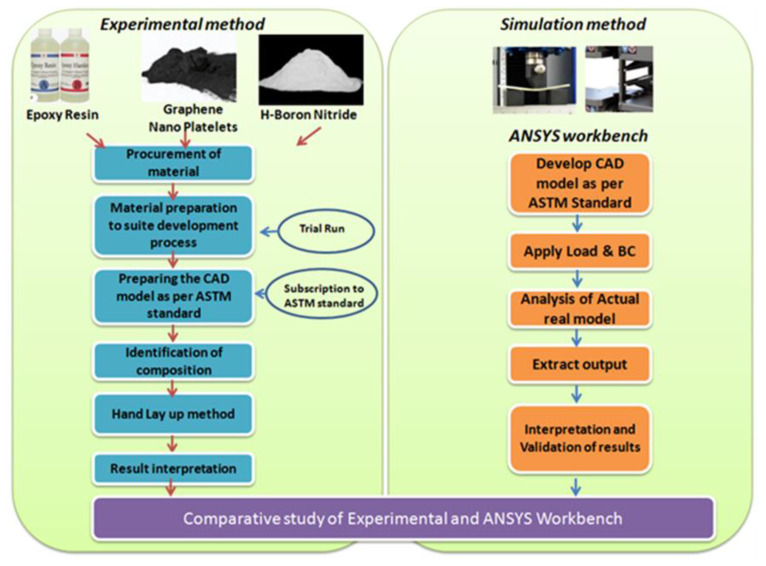
Process map for simulation vs. experimental work.

Among the simulation software, ANSYS workbench is currently the leading tool in the industry and can solve multidisciplinary problems. The roadmap for solving this current problem has been considered with the following process map, as shown in Figure 10. Figure 10 discusses the experimental and simulation study in the stepwise process. The entire study ends with a comparative study between the experiment and simulation. The work in the experiment initiates with the procurement of raw materials such as epoxy resin, h-BN and GNP. Then, it involves preparing the raw material to be ready for specimen development and further maintaining the specimen shape and size as per ASTM standards. Afterwards, there is the finalisation of the composition for the hand lay-up method and testing in micro UTM. On the other side, simulation work was carried out in the ANSYS workbench with input parameters such as material property, loads and boundary conditions resulting in deformation and stress values depending on the test conditions, either tensile or flexural. These outcome parameters were compared with experimental results, and an optimal model was re-developed physical and tested for final validation.

#### 4.1.1. Simulation Process

The current simulation tool deals with the flexural strength test geometry model, leading to deformation estimation [33]. For better correlation purposes, one case with PE + 0.6 wt % of GNP was considered with several iterations to converge the solution along with validation. 

#### 4.1.2. Material Properties

Details of the material properties of the h-BN and GNP, along with epoxy resin, are shown in Table 11.

#### 4.1.3. Geometry

**Figure 11:**
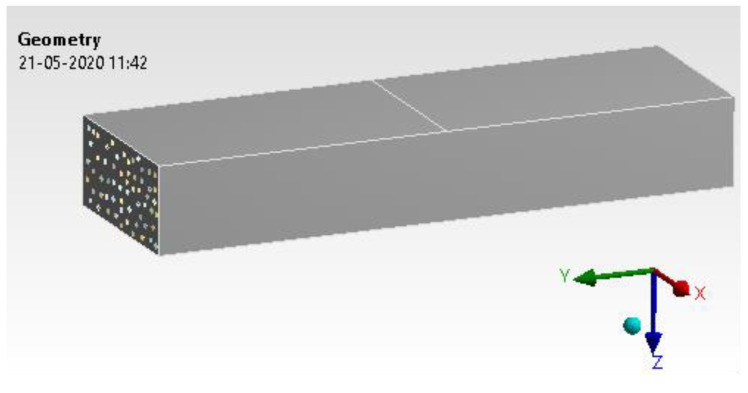
Epoxy and h-Boron Nitride/Graphene.

As mentioned in the earlier section, the CAD model was developed with a suitable size and shape. The model is shown in Figure 11. The entire CAD model is divided into three main clusters; firstly, the main substrate is made out of epoxy resin with a specimen size of 135 mm × 15 mm × 6 mm. However, h-BN and GNP are used separately and built as per a weight ratio of 0.6%. These are built randomly in the entire substrate, matching the weight fraction of the epoxy resin. The focus is to match the composition by weight fraction with h-BN and GNP. The h-BN and GNP cluster is oriented, assuming they will have random directions when mixed in real time before solidification. An ideal condition is arrived at based on the number of iterations to mimic this orientation behaviour. The convergence study is carried out to correlate the compatibility equations with the H-type and P-type methods. The focus here is to reduce the number of experiments by achieving the optimal condition in the dispersion process [34].

#### 4.1.4. Contact Generation

**Figure 12:**
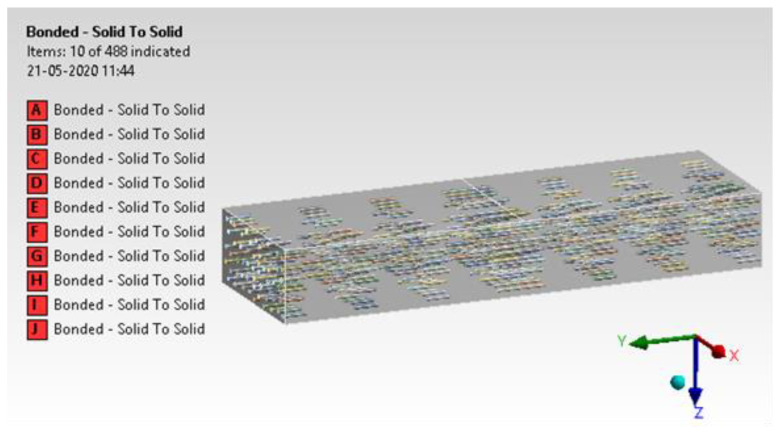
Contact generation.

Contact generation between h-BN/GNP and epoxy is assigned with ‘Bonded’ contact. Each of these contacts was considered with a ‘Pure penalty’ approach [35,36]. The details of contact generation are illustrated in Figure 12.

#### 4.1.5. Mesh Generation

**Figure 13:**
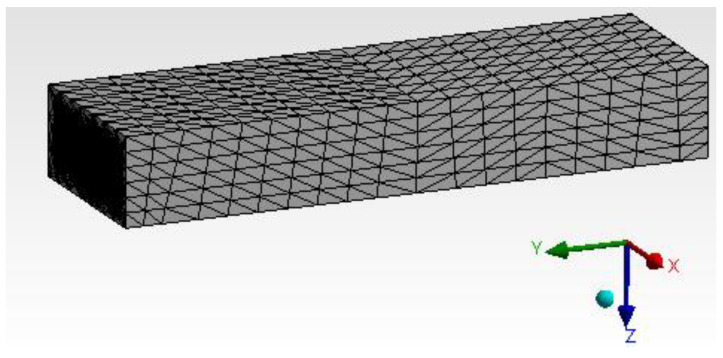
Mapped face meshing.

Mesh generation has been assigned with mapped face meshing to arrive at the exact solution. The H-type and P-type methods were used to analyse the results. The process uses a tetrahedron element with ten nodes of the second-order condition [37]. From Figure 13, it provides satisfactory mesh conditions while checking other converging conditions. The entire model was solved for 326,363 elements and 857,067 nodes.

#### 4.1.6. Loads and Boundary Conditions

**Figure 14:**
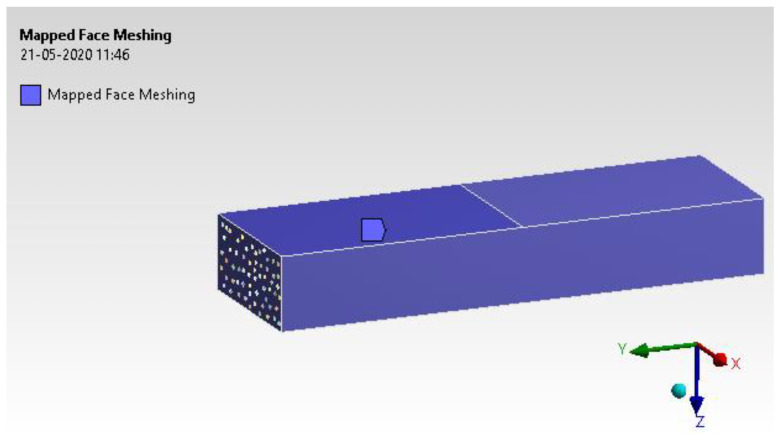
Mapped face meshing.

The details were fetched from experimental analysis to arrive at loads and boundary conditions. The three-point bend test is considered with displacement free in the y-direction, while the other two directions were fixed. Figure 14 illustrates the loading details and boundary conditions.

#### 4.1.7. Results and Interpretation

**Figure 15:**
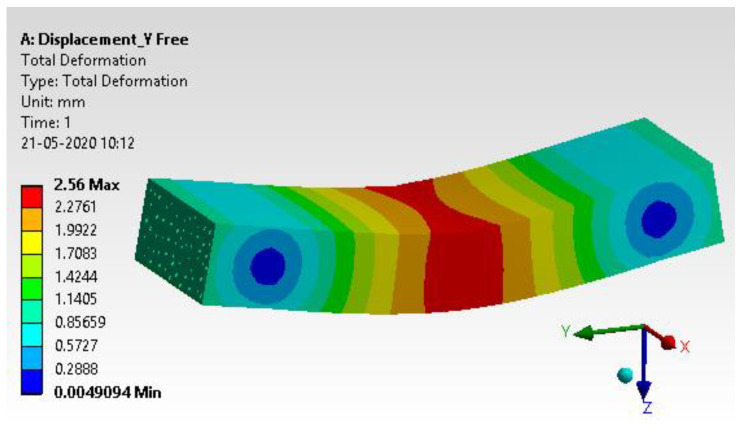
Total Deformation.

The total deformation has been extracted from the analysis, and details are discussed in Figure 15 [38]. The total deformation observed is 2.56 mm, which is more than 16% higher than the experimental work, but for a typical composite material, the percentage difference should be below 20%. This validates the aforementioned results.

#### 4.1.8. Comparative Study with Validation

Experimental method results were then compared with simulation results with tabular data, as shown in Table 12. The comparative study reveals a percentage error of 16.36, which is well accepted by an industry standard for a composite material; the acceptable error range is 20% [39,40,41].

### 4.2. h-Boron Nitride and Graphene with Polyimide Substrate

**Figure 16:**
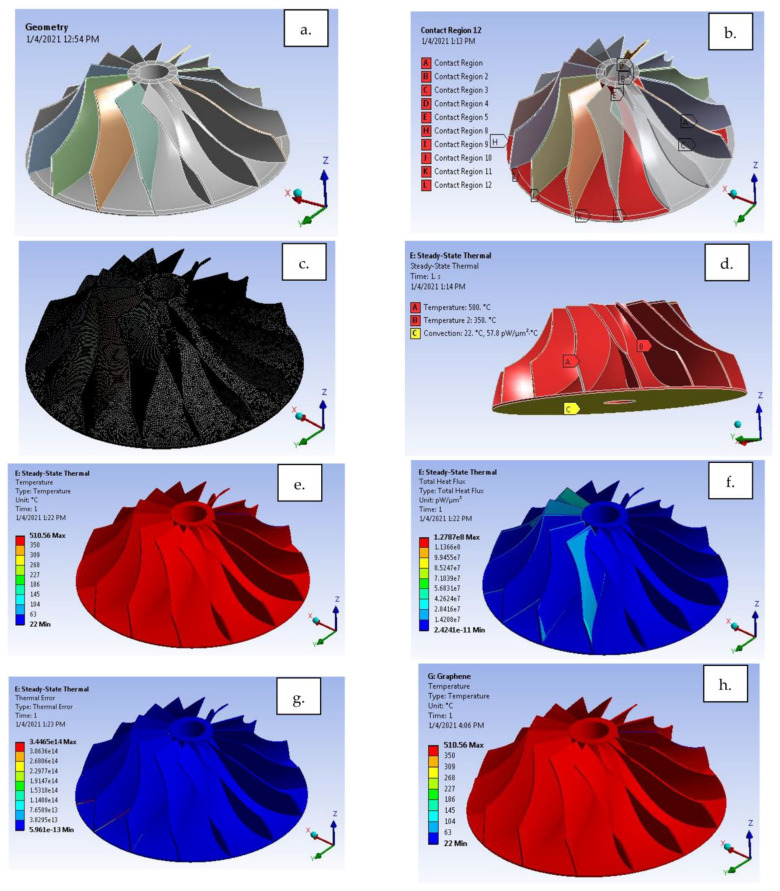

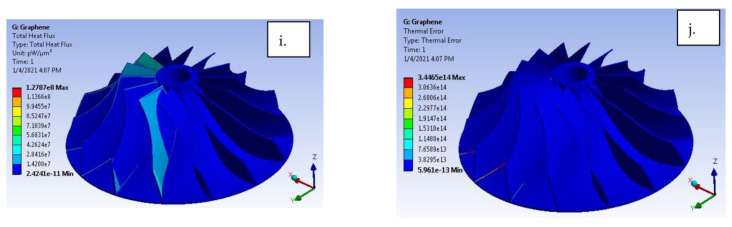
Compressor blade (**a**) Geometry, (**b**) Contact generation, (**c**) Mesh generation, (**d**) Loads and boundary conditions, (**e**) Temperature gradient for h-BN coating, (**f**) Heat flux, (**g**) Thermal error, (**h**) Temperature gradient for graphene coating, (**i**) Heat flux, (**j**) Thermal error.

A compressor blade with h-BN and GNP coatings is used to validate the results with polyimide as the substrate. The current application is derived from a gas turbine engine compressor blade. Furthermore, details of the simulation are discussed in Figure 16a–g.

The application selected is based on the subject of temperature, torque and boundary conditions. A similar kind of work on compressor blades was observed in the case of rigid viscoelastic compressor blades by Zhan et al. [42], with strain values 0.22 to 0.47 for given loads and boundary conditions. Temperature gradient and heat flux-based works are scantly reported. In contrast, a structural analysis of compressor blades for gas turbine blades observed a stress field in the blade in the vicinity of t = 0.1 s where bending motions are dominant [43]. In another work, safe fatigue life reduces by about 32% in the case of 8B (rotor blade design) and 40% in the case of 8C (rotor blade design) compared with conventional approaches where aerodynamic forces are omitted [44]. All these are typical cases observed for compressor blades at various loads and boundary conditions.

From Figure 16b, it can be inferred that ‘Bonded’ contact is generated between the blade and base plate as a weldment. The simulation carried out for graphene combination shows a total heat flux of $1.27\times 10^{8}$ W/μm^2^. The temperature was raised from 500 to 510 °C for both GNP and h-BN material, making it a non-thermal material to dissipate heat comparatively higher than other thermal barrier materials currently in use.

## 5. Conclusions

The experimental and simulation research indicated that the novel polymer composites reinforced with GNP and h-BN showed better performance than plain epoxy composites. The main conclusions drawn follow.

It was observed that an increase in filler material concentration resulted in an increased tensile property. Still, this nature was observed only up to 0.2 weight percent of the epoxy composite reinforced with GNP, which was followed by a gradual decrease with a higher dosage of GNP. The properties of epoxy composite reinforced with GNP and h-BN increased even at a 0.6 weight percentage. This nature may be because of the nanoparticle’s uniform distribution in the epoxy matrix, as shown in SEM analysis. The composite’s load-bearing capacity with GNP increased by 265% compared with the plain epoxy composite. The composite’s load-bearing capacity with GNP and h-BN increased by 219% compared with the plain epoxy composite. 

TGA revealed that the GNP and h-BN reinforced epoxy composite hindered the mass degradation regarding elevated temperature. The material degradation for plain epoxy composites was started at a temperature of around 242 °C. However, the material degradation for epoxy composites reinforced with GNP and GH was started at a temperature of around 340.99 °C and 342.43 °C, respectively. In other words, the initial 1.3% material degradation was enhanced by 40.9% and 41.5% compared to plain epoxy composite.

The numerical simulation using ANSYS workbench to reach the optimal composition endorsed a small error in experiments, less than 20% error, and more than 83% confidence level.

Hence, the novel proposed polymer nanocomposite reinforced with GNP and h-BN can be used for high-temperature applications around 340 °C. This composite with reduced weight and increased mechanical strength can substitute for ceramic-based composites.

## Figures and Tables

**Table 1:** Properties of the GNP and h-BN used in this study.

Specifications	Dimensions (GNP)	Dimensions (h-BN)
Diameter	2–4 (nm)	60 (nm)
Purity	99.5 (%)	99.9 (%)
Density	2.3 (g/cm^3^)	2.29 (g/cm^3^)
Molecular Weight	12.01 (g/mol)	24.82 (g/mol)
Young’s Modulus	1 (TPa)	3.5 (TPa)
Melting Point	>3600 (°C)	2527 (°C)

**Table 2:** Test specimen details (Epoxy + GNP).

No.	Specimen Reference	Elements	Percentage of GNPs by wt
1	PE	Plain epoxy	0.0
2	GNP1	Plain epoxy + GNPs	0.1
3	GNP2	Plain epoxy + GNPs	0.2
4	GNP3	Plain epoxy + GNPs	0.3
5	GNP4	Plain epoxy + GNPs	0.4
6	GNP5	Plain epoxy + GNPs	0.5
7	GNP6	Plain epoxy + GNPs	0.6

**Table 3:** Test specimen details (GNP and h-BN).

Sample No.	Specimen Reference	Constituents	Percentage of GNP and h-BNs by wt
1	PE	Plain epoxy	0.0
2	GH1	Plain epoxy + GNPs + h-BN	0.1
3	GH2	Plain epoxy + GNPs+ h-BN	0.2
4	GH3	Plain epoxy + GNPs+ h-BN	0.3
5	GH4	Plain epoxy + GNPs+ h-BN	0.4
6	GH5	Plain epoxy + GNPs + h-BN	0.5
7	GH6	Plain epoxy + GNPs+ h-BN	0.6

**Table 4:** Sample Characteristics (Tensile).

No.	Parameters	Details
1.	Number of samples tested per combination	04
2.	Specimen size	165 mm × 19 mm × 6 mm
3.	Epoxy resins	L-12
4.	% of GNP/h-BN	0.1, 0.2, 0.3, 0.4, 0.5 and 0.6% by weight of epoxy resin

**Table 5:** Sample Characteristics (Flexural Test).

No.	Parameters	Details
1.	Number of samples tested per combination	04
2.	Specimen size	135 mm × 15 mm × 6 mm
3.	Epoxy resin	L-12
4.	% of GNP/h-BN	0.1, 0.2, 0.3, 0.4, 0.5 and 0.6% by weight of Epoxy resin.

**Table 6:** Sample Characteristics (Four-Point Bending).

No.	Parameters	Details
1.	Number of samples tested per combination	04
2.	Specimen size	230 mm × 13 mm × 6 mm
3.	Epoxy resin	L-12
4.	% of GNP/h-BN	0.1, 0.2, 0.3, 0.4, 0.5 and 0.6% by weight of epoxy resin

**Table 7:** Specification of SEM.

Parameters	Details
Instrument make Resolution	JEOL JSM-63OLA 4 nm
Electron gun	Tungsten filament

**Table 8:** Specification of EDX attachment.

Parameters	Details
Instrument make Acc. voltage	6380(LA) 20.0 kV
Probe current	1.00000 nA

**Table 9:** Different species present in 0.4 wt % of h-BN in epoxy.

Element	Weight %
B	4.20
N	8.44
C	68.32
O	18.62

**Table 10:** Different species present in 0.6 wt % of GNP and h-BN in epoxy.

Element	Weight %
B	4.89
N	15.09
C	47.52
O	32.50

**Table 11:** Material properties for h-BN and GNP.

Sl. No.	Material	Young’s Modulus, MPa	Poisson’s Ratio	Density (kg/m^3^)
1	Epoxy resin	130 × 10^3^	0.33	1400
2	h-BN	16 × 10^3^	0.24	2100
3	GNP	1 × 10^6^	0.3	2300

**Table 12:** Comparative study.

Description	Experimental Method	Simulation Method	% of Error
Total deformation (in mm)	2.2	2.56	16.36

## Data Availability

The data presented in this study are available upon request from the corresponding author.
